# Drought and plant neighbourhood interactively determine herbivore consumption and performance

**DOI:** 10.1038/s41598-018-24299-x

**Published:** 2018-04-12

**Authors:** Bastien Castagneyrol, Xoaquín Moreira, Hervé Jactel

**Affiliations:** 1 BIOGECO, INRA, Univ. Bordeaux, 33610 Cestas, France; 2 Misión Biológica de Galicia (MBG-CSIC), Apartado de correos 28, 36080 Pontevedra, Galicia Spain

## Abstract

Both plant neighbourhood composition and drought have well-known independent effects on insect herbivore performance, but their interactive effects remain elusive. In this study we performed a laboratory experiment to investigate the independent and combined effects of plant neighbourhood composition and drought on the performance of Gypsy moth larvae (*Lymantria dispar*) feeding on silver birch (*Betula pendula*) leaves. For this, we collected leaf samples from birch trees growing in a field experiment where we manipulated both host-tree species diversity (three levels: birch monocultures, two-species mixtures associating birch with the pedunculate oak *Quercus robur* or maritime pine *Pinus pinaster*, and three-species mixture with pedunculate oak, the maritime pine and birch) and water availability (two levels: irrigated vs. non-irrigated). In most cases, plant neighbourhood composition and irrigation treatments independently and interactively affected herbivore performance traits, especially those related to growth and food (i.e. birch leaves) processing. By addressing the interactive effects of tree species diversity and drought on insect herbivory from the herbivore’s point of view, our study builds toward a better understanding of the multiple ecological drivers of plant-insect interactions.

## Introduction

A rich body of concepts has been developed to predict the effects of plant diversity, measured as the number of plant species or genotypes within a given area, on insect herbivore communities and the mechanisms underpinning such effects^1^. In particular, the concepts of associational resistance or susceptibility have been proposed, where the presence of neighbouring plants of the same vs. different species (or genotypes) modifies herbivore foraging behaviour and consumption of a given focal plant^2^. For instance, the “Resource Concentration Hypothesis” posits that herbivores frequently forage in a density-dependent manner, and therefore increasing number of plant species or genotypes at a constant plant density may reduce the probability of finding a preferred host plant species (or genotype) by herbivores, ultimately leading to lower herbivore abundance and damage on individual plants^3,4^. Moreover, associational effects may be generated by plant neighbours modifying host apparency and therefore plant likelihood of being detected by herbivores^5,6^. Alternatively, plant neighbourhood diversity might indirectly affect herbivory on focal plants by modifying plant nutritional quality (e.g., physical traits and secondary metabolites), independently of resource concentration or associational effects^7–12^. For example, competition for resources or facilitation among heterospecific plants or changes in abiotic conditions may alter plant growth or the nutritional value of plant tissues to herbivores^13^. Last the diversity of plants may offer a larger array of feeding resources or shelters to natural enemies controlling herbivore population^14^.

A widely used approach by ecologists and entomologists interested in associational effects consists in measuring percentage of leaf area removed by chewing herbivores or the proportion of leaves with specific damage such as those inflicted by leaf-miners or gall-makers^5,8,15–17^. However, by exclusively focusing on associational effects from the plant’s point of view, one may not fully understand the underlying mechanisms because any effect of plant diversity on defoliation may result from plant diversity correlating with herbivore diversity or abundance^1,2^ and/or from changes in herbivore feeding behaviour (i.e., once herbivores have reached and start consuming plants). To gain more insights, we need to also take into account the herbivore’s point of view, for example by estimating herbivore performance on plants growing in the presence of conspecific or heterospecific neighbouring plants^18,19^.

The study of plant diversity effects on plant-herbivore interactions have commonly been addressed in a single environment, neglecting the potential interacting effects of plant diversity and abiotic factors on herbivores. It is well known that drought can affect herbivore performance and consumption rates by altering plant defences or the nutritional value of plant tissues^20–22^. Along these lines, a meta-analysis by Jactel *et al*.^23^ suggested that defoliators benefit from drought as a likely result of increased concentration of free sugars and amino acids in leaf cells of the stressed plants. In addition, plant diversity may attenuate the way by which focal plants respond to water stress^24–26^. For instance, Klaus *et al*.^27^ showed that plant diversity reduced the negative impact of drought on biomass production by grasses and legumes, as a likely result of complementarity in root depth and biomass^28^. Any effect of plant diversity on plant response to drought may thus have cascading effects on herbivores. Recently, some promising studies addressed the mechanisms by which plant neighbourhood and abiotic factors may interactively drive insect herbivory^9,22,29,30^, showing interactive effects of plant diversity and climatic factors on plant defences or insect abundance and damage. However, our knowledge on this topic still remains scarce, particularly in forest systems.

In this study we performed a laboratory experiment to investigate the independent and interactive effects of plant neighbourhood composition and drought on the performance (i.e. nutritional indices) of a generalist insect herbivore (the Gypsy moth *Lymantria dispar* L., Lepidoptera) feeding on silver birch (*Betula pendula*, Betulacae) leaves. For this, we collected leaf samples from birch trees growing in a field experiment where we manipulated both species composition of birch neighbours (four levels: birch monocultures, two-species mixtures associating birch with the pedunculate oak *Quercus robur* or the maritime pine *Pinus pinaster*, and three-species mixtures associating birch with the pedunculate oak and the maritime pine) and water availability (two levels: irrigated vs. non-irrigated). By addressing associational effects from an herbivore’s point of view, this study advances our mechanistic understanding of the interactive effects of plant species diversity and abiotic conditions on plant-herbivore interactions.

## Results

### Gypsy moth consumption and performance in the laboratory

#### Leaf consumption

**Figure 1:**
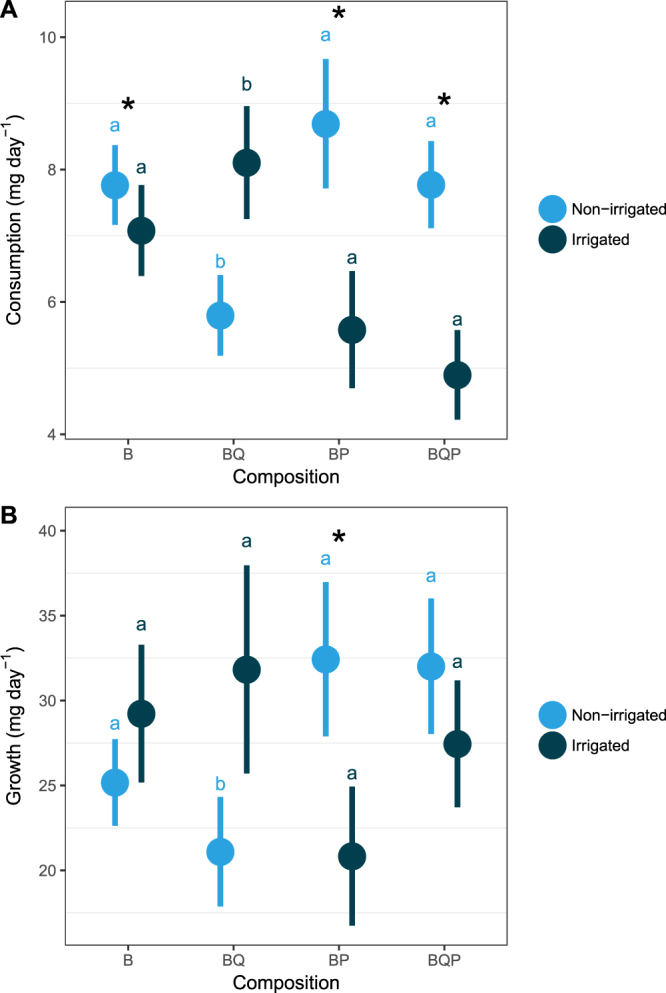
Effects of tree neighbourhood composition and irrigation on *Lymantria dispar* larval consumption (**A**) and growth (**B**) on birch leaves in the laboratory. Although models were based on log-transformed variables, dots and error bars represent raw means and corresponding standard errors. Contrasts among composition treatments were estimated for each level of irrigation separately. Contrasts among irrigation treatments were estimated for each composition separately. Same letters above bars indicate non significant differences among composition treatments. Black stars indicate significant differences among irrigation treatments.

Under laboratory conditions, gypsy moth larvae consumed on average (±SD) 6.96 ± 2.83 mg day^−1^ of birch leaves. There was no significant effect of tree species composition, *per se*, on leaf consumption (Table 1). Irrigation treatment (but not tree species composition) significantly affected larval consumption (ΔB, Table 1). Specifically, larvae consumed 1.2-fold more amount of leaf material from trees growing in non-irrigated plots (7.42 ± 2.22 mg day^−1^) than from trees growing in irrigated plots (6.40 ± 2.77 mg day^−1^, Fig. 1A). However, herbivore consumption patterns largely varied between different tree neighbourhood compositions, depending on the irrigation treatment as demonstrated by the significant Irrigation × Composition interaction (Table 1). In particular, leaf consumption was significantly greater in non-irrigated plots than in irrigated plots in all tree species composition treatments, except in birch-oak mixtures where irrigation had no significant effect (Fig. 1A). Leaf consumption significantly increased with increased larval initial weight (coefficient parameter estimate ± SE: 0.035 ± 0.005, Table 1). However, the interactive effect of Irrigation × Composition treatments on leaf consumption remained significant after including initial weight as a covariate in the statistical model (Table 1), indicating the existence of pre-ingestive regulatory processes.

**Table 1:** Summary of ANCOVA models testing the effects of tree neighborhood composition and irrigation on nutritional ratios of Lymantria dispar larvae feeding on birch leaves.

**Nutritional ratio equivalent**	**Response variable**	**Predictor**	**F**(**df**)	**P-value**	**R²**
Relative Consumption Rate (RCR)	Consumption (ΔB)	w_i_	40.77 (1, 86)	**<0**.**001**	0.48
		Irrigation	25.55 (1, 86)	**<0**.**001**	
		Composition	0.83 (3, 86)	0.480	
		Irrigation × Composition	8.66 (3, 86)	**<0**.**001**	
Relative growth rate (RGR)	Growth (w_f_ − w_i_)	w_i_	43.46 (1, 85)	**<0**.**001**	0.36
		Irrigation	3.11 (1, 85)	0.082	
		Composition	1.34 (3, 85)	0.267	
		Irrigation × Composition	2.88 (3, 85)	**0**.**041**	
Efficiency of conversion of ingested food (ECI)	Growth (w_f_ − w_i_)	Consumption	285.52 (1, 81)	**0**.**003**	0.78
		Irrigation	9.65 (1, 81)	**0**.**015**	
		Composition	6.18 (3, 81)	**<0**.**001**	
		Consumption × Irrigation	6.75 (1, 81)	**0**.**011**	
		Consumption × Composition	3.72 (3, 81)	**0**.**015**	
		Irrigation × Composition	5.79 (3, 81)	**0**.**001**	
Approximate digestibility (AD)	Frass	Consumption	342.35 (1, 83)	**<0**.**001**	0.82
		Irrigation	35.86 (1, 83)	**<0**.**001**	
		Composition	3.46 (3, 83)	**0**.**020**	
		Consumption × Irrigation	15.55 (1, 83)	**<0**.**001**	
		Consumption × Composition	4.57 (3, 83)	**0**.**005**	
		Irrigation × Composition	9.14 (3, 83)	**<0**.**001**	
Efficiency of conversion of digested food (ECD)	Growth (w_f_ − w_i_)	Digested (Consumption - Frass)	10.41 (1, 81)	**0**.**002**	0.27
		Irrigation	2.47 (1, 81)	0.120	
		Composition	1.02 (3, 81)	0.388	
		Digested × Irrigation	6.13 (1, 81)	**0**.**015**	
		Digested × Composition	3.08 (3, 81)	**0**.**032**	
		Irrigation × Composition	5.21 (3, 81)	**0**.**002**	

For each model, response and explanatory variables are indicated, together with the nutritional index they correspond to (see Fig. 1). Non-significant interactions were sequentially removed from the model starting with the least significant ones. We report statistics and P-values for the reduced, final model. w_i_: initial weight, w_f_: final weight, ΔB: leaf biomass consumed by gypsy moth larvae. R² are given for the simplified model. Characters in bold font refer to significant effects at P < 0.05.

#### Growth

Although tree species composition and irrigation treatments did not significantly affect the relative growth rate of gypsy moth larvae (i.e., no significant w_i_ × Irrigation or w_i_ × Composition interactions, Table 1), we found a significant effect of the Irrigation × Composition interaction on herbivore absolute growth (Table 1). In particular, gypsy moth larvae grew significantly more when feeding on leaves from non-irrigated birch-pine mixtures than when they fed on leaves from irrigated birch-pine mixtures (Fig. 1B). Contrarily, larval growth did not significantly differ among irrigation treatments for the other tree neighbourhood compositions (Fig. 1B). Gypsy moth larvae fed leaves from non irrigated plots grew less when leaves were taken from birch-oak mixtures than from any other neighbourhood (Fig. 1B). At the contrary, there were no significant differences in gypsy moth larvae growth among tree neighbourhood for leaves taken from irrigated plots (Fig. 1B).

#### Efficiency of conversion of ingested (ECI) and digested (ECD) food into body mass

Overall, larval growth increased with the amount of food (i.e., leaves) consumed (coefficient parameter estimate ± SE: 3.49 ± 0.73, Table 1, **ECI**) and digested (1.49 ± 2.20, Table 1, **ECD**).

**Figure 2:**
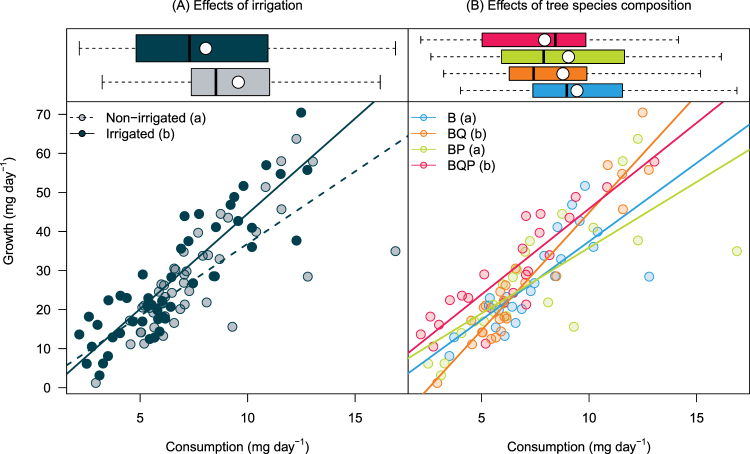
Efficiency of conversion of ingested food into body mass (ECI) by *Lymantria dispar* larvae feeding on birch leaves. Dots represent raw data. ECI corresponds to the slope of regression lines. Boxplots in upper panels indicate variability of consumption among (**A**) composition and (**B**) irrigation treatments. In boxplots, dots and vertical bars indicate treatment mean and median, respectively. Significant differences among slopes are indicated by different lower case letters in legends. B: Birch, BQ: Birch + Oak, BP: Birch + Pine, BQP: Birch + Oak + Pine.

ECI differed among Irrigation and Composition treatments as indicated by the significant Consumption × Irrigation and Consumption × Composition interactions (Table 1, **ECI**). ECI was significantly greater for larvae feeding on leaves from irrigated plots than for those feeding on leaves from non-irrigated plots (Fig. 2A), indicating that the same growth could be achieved with lower consumption in irrigated plots (*i*.*e*., steeper slope). ECI was significantly greater for larvae feeding on leaves from birch-oak and birch-oak-pine mixtures (*i*.*e*., steeper slope) than for those feeding on leaves from birch monocultures or birch-pine mixtures (Fig. 2B).

The effects of irrigation and tree species composition on ECD were qualitatively and quantitatively comparable to those reported for ECI (Table 1, **ECD**), indicating that differences in larval growth between irrigation and composition treatments resulted from both pre-ingestive (*i*.*e*., differential consumption, ECI) and post-digestive (*i*.*e*., metabolic, ECD) processes.

#### Approximate digestibility (AD)

Larvae produced on average (±SD) 3.89 ± 1.90 mg_frass_ day^−1^. Frass production significantly increased with leaf biomass consumption in all treatments (coefficient parameter estimate ± SE: 0.48 ± 0.08, Table 1, AD). For a given consumption, the lower frass production, the more digestible the food, thus the flatter the regression slope between frass production and consumption. Larvae produced 1.1 times more frass when fed on leaves from irrigated plots (4.07 ± 2.12 mg day^−1^) than when they fed on leaves from non-irrigated plots (3.71 ± 1.65 mg day^−1^). Frass production varied by less than 6% among composition treatments, ranging from 3.78 ± 1.70 mg day^−1^ when larvae were fed on leaves from birch monocultures to 3.99 ± 2.32 mg day^−1^ when they fed on leaves from birch-oak mixtures.

**Figure 3:**
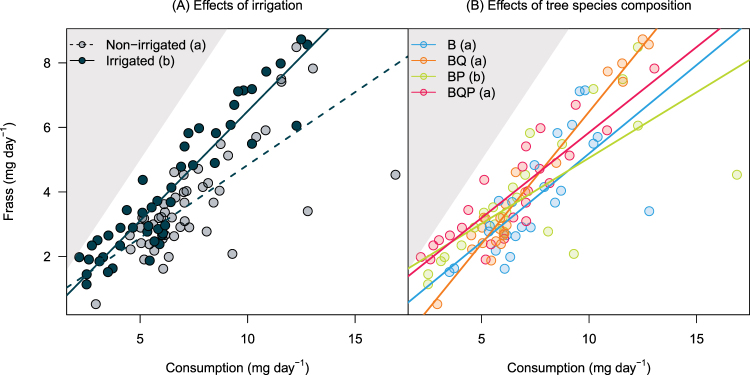
Approximate digestibility (AD) of birch leaves processed by Lymantria dispar larvae. Dots represent raw data. AD corresponds to regression slopes divided by 1. Significant differences among slopes are indicated by different lower case letters in legends. The steeper the slope, the lower the digestibility. In each panel, grey areas indicate the impossible case above the 1:1 line, that is higher frass production than leaf consumption. B: Birch, BQ: Birch + Oak, BP: Birch + Pine, BQP: Birch + Oak + Pine.

The effects of Irrigation and Composition treatments on frass production were significant (Table 1) but were contingent upon consumption as indicated by the significant Consumption × Irrigation and Consumption × Composition interactions (Table 1, **AD**). AD of leaves taken from non-irrigated was significantly greater than AD of leaves from irrigated plots (i.e., flatter slope in Fig. 3A). AD was significantly greater for leaves from birch-pine mixtures than from any other plots (Fig. 3B). These results indicated that differences in larval absolute growth between composition and irrigation treatments partly resulted from differences in birch leaf nutritional quality for gypsy moth larvae.

## Discussion

Our results showed that under laboratory conditions performance of gypsy moth larvae feeding on birch leaves markedly varied among tree species composition treatments, with a better food conversion in mixtures where birch was associated with oak. However, this significant effect was contingent upon tree water-stressed conditions. In particular, such increased herbivore performance in oak-birch mixtures was only observed under drought conditions. Together, these findings bring new insights into our understanding of ecological factors responsible for variability in leaf insect herbivory and associational effects in mixed forests^1,30^.

### How gypsy moth larvae processed food was dependent on tree species composition

Our results showed that performance of gypsy moth larvae on birch leaves markedly varied among tree species composition treatments. In particular, ECI and ECD were greater (i.e., larvae grew more for the same consumption and digestion) on birch growing in birch-oak mixtures (and to a lower extent in birch-oak-pine mixtures) than in birch monocultures and birch-pine mixtures. It is likely that tree species composition altered leaf quality (and ultimately gypsy moth performance) through changes in the light environment around birch leaves^19,31^. In our experiment, birch height was intermediate between that of oaks (small) and pines (tall) such that birch leaves were more sun exposed in birch-oak mixtures, and more shaded in birch-pine mixtures than in monocultures and three-species mixtures^6,32^. However, only a further assessment of chemistry in terms of primary and secondary metabolites, as well as physical defences, would give insights about mechanisms responsible for observed differences in gypsy moth performance among composition treatments^13,33,34^.

**Figure 4:**
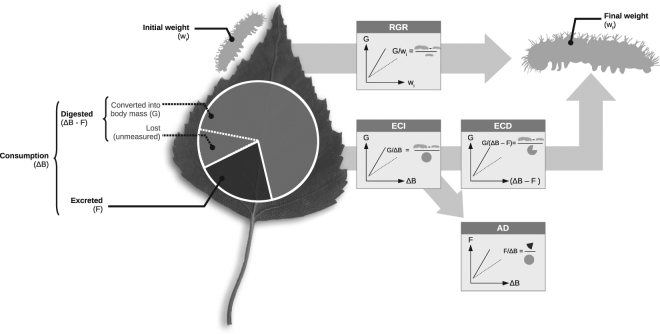
Schematic representation of nutritional indices and their ANCOVA equivalents. For each nutritional index (shown in bold font, **AD**: Approximate Digestibility, **ECI**: Efficiency of Conversion of Ingested food, **ECD**: Efficiency of Conversion of Digested food, **RGR**: Relative Growth Rate), formula and ANCOVA equivalents are shown. The ANCOVA equivalent of AD takes Frass as a response variable and Consumption as a covariate such that AD is given by 1 ÷ (F/ΔB). ΔB: leaf biomass consumption, F: Frass production, G: Larval growth (w_f_ − w_i_).

ECI and ECD were lower in birch-pine mixtures suggesting that gypsy moth larvae were not fulfilling their need in macronutrients when fed on this diet. In sharp contrast, we found that larvae produced less frass from the same amount of ingested food when feeding on birch leaves from birch-pine mixtures than when feeding on birch leaves from plots of other composition. This apparent contradiction between greater digestibility but lower efficiency of food conversion may result from gypsy moth larvae adapting post-ingestive but not pre-digestive regulatory processes to compensate for lower food quality^35^. Alternatively, this could be due to a higher proportion of digested biomass that was actually lost as a result of differential enzymatic activity after digestion^35^, which could not be accounted for in the present study (Fig. 4).

Our results also found no overall effect of tree species composition on gypsy moth larval growth or consumption. This finding runs counter to previous studies reporting that leaf insect herbivory is strongly affected by tree diversity, and in particular by the composition of tree species neighbourhood^1^. It is important to note that in our study we only measured leaf insect herbivory by one generalist herbivore for a short period of time. Further studies in this system should include the entire herbivore community and over longer time periods to generalize our patterns. In addition, although *Lymantria dispar* is a generalist herbivore, we cannot guarantee that the present findings obtained on birch leaves would have been comparable on another host species such as the pedunculate oak.

Despite these limitations, our study is one of the few to date that has addressed the effects of plant species neighbour composition on herbivory from an herbivore point of view, *i*.*e*., focusing on feeding of herbivores instead of assessing herbivore damage in plants^18,19,36^. In one of the few available studies, Alalouni *et al*.^18^ found that tree species richness did not significantly influence growth or consumption of the gypsy moth larvae feeding on oaks (*Quercus robur*) in the laboratory, despite they observed a significant negative effect of tree species richness on leaf damage in the field, which they attributed to a greater variability of leaves within a tree than between trees in different tree species richness levels. These apparent discrepancies stress the need to further address simultaneously leaf damage and different aspects of herbivore performance to better understand tree diversity and composition effects on herbivory.

### Gypsy moth larvae performed better on stressed trees

We found that, overall, gypsy moth larvae consumed more leaf material when fed on leaves from non-irrigated plots and that their efficiency of conversion of food (both ECI and ECD) were lower in non-irrigated plots. However, growth rate of gypsy moth larvae was similar in irrigated and non-irrigated plots. These results suggest that gypsy moth larvae compensated for the poor quality of leaves from non-irrigated plots (as indicated by lower ECI and ECD) by consuming more biomass, which corresponds to pre-ingestive regulatory processes. In addition, AD (*i*.*e*., an index reflecting post-ingestive, pre-digestive processes) was greater in non-irrigated plots, which suggests that post-ingestive regulatory processes (where ingested food is processed in the midgut) were equally at play to compensate for lower food quality.

We did not find a consistent effect of irrigation on growth of gypsy moth larvae. Although this is in line with the observation that, among insect herbivores, chewing herbivores are those less affected by water stress^21^, this does not imply that irrigation had no effect on herbivory. At the contrary, we found that (i) gypsy moth larvae adapted their consumption and metabolic activity to drought conditions of their host trees and (ii) gypsy moth larvae consumed more leaf biomass to compensate for lower food quality. From a tree perspective, this would imply a greater amount of damage in drought conditions^23^. Future studies addressing herbivore response to drought should disentangle the effects of drought on various aspects of both plant quality (including both primary and secondary metabolites and constitutive vs. induced defences) and herbivore metabolic activity.

### Water stress altered the effect of tree species composition on gypsy moth performance

The effects of tree species composition on gypsy moth larvae consumption and growth on birch leaves were contingent upon water stress. In particular, we found that gypsy moth consumption and growth on birch growing within birch-oak mixtures was lower than in other composition treatments, but only in non-irrigated plots. One plausible explanation for these findings is that the presence of smaller oaks has modified the effect of drought on birches. On the one hand, being of intermediate height between oaks and pines, birch may have received more light, grew faster, increasing the need for water and thus being more water-stressed in birch-oak mixtures than in monocultures or in mixtures with pines. On the other hand, birch trees may have faced less inter- and intra-specific competition of water when associated to small oaks as compared to monocultures and birch-pine mixtures. Although the precise mechanism through which tree species composition affected the interaction between birch trees and gypsy moth larvae cannot be unravelled with the present data, it is likely that the effect of plant neighbourhood on drought conditions drastically modified resource allocation patterns in plants (and in turn leaf quality), as shown for herbaceous^22^ and woody^36–38^ plant species. For instance, Hommel *et al*.^38^ found that water-stressed beeches allocated more photoassimilates to roots, and even more in mixtures with maple, which may greatly reduce its ability to invest in leaf defences. However, in previous studies using the same experiment, we found no such interactive effects of drought and tree species composition treatments on several oak or birch leaf traits such as leaf surface, leaf dry matter content or polyphenolics^32,39^. These results suggest that lower growth in non-irrigated birch-oak mixtures was primarily driven by a lower consumption. Altogether, our results indicate that tree diversity operates on insect herbivores via different mechanisms that can be only fully understood by simultaneously addressing the responses of plant functional traits, herbivore performance and feeding behaviour.

### Limitations of this study

Caution is needed when extrapolating results from laboratory feeding trials to herbivory in the real world. First, herbivores were reported to adapt their feeding behaviour to leaf quality and in particular to the induction of anti-herbivore defences. For instance, many herbivores quickly abandon induced leaves and move on to non-induced parts of a plant^40^. Short-term non-choice feeding experiments may fail to capture the effect of variability in leaf quality and in particular the effects of induced defences that may take time to be mounted. In addition, we cannot exclude that leaf excision induced defences that made leaf quality different in the laboratory as compared to the field experiment. Although the same approach was applied to all experimental treatments, it is possible, in principle, that tree diversity and/or drought affected tree ability to induce defences. This possibility will require further investigations in the future. Second, laboratory feeding trials do not include the third trophic level, which may overestimate herbivore performance, in particular because herbivores where shown to adapt their feeding behaviour and metabolism to predation risk^41,42^. Despite these limitations, it is important to note that because we conducted our experiment in laboratory conditions where only food quality differed among treatments such that differences in herbivore consumption and growth were not affected by herbivore foraging decisions. Therefore, our results suggest that the interactive effects of tree diversity and irrigation on insect herbivory partly results from both neighbour-mediated changes in leaf quality and changes in herbivore feeding processes, and not only from differences in herbivore’s host searching behaviour as commonly inferred from field studies^6,17,19^.

### Conclusions

Our study adds to the growing body of evidence that tree species diversity influences insect herbivory, i.e., the amount of plant biomass removed by herbivores. Herbivory is determined by both the interplay between plant quality and defences and the herbivore behaviour and physiology. By addressing tree diversity effects on both aspects of herbivory, we provide new insights into our understanding of mechanisms at play. First, we demonstrate that herbivore performance on a given tree are partly mediated by the indirect, trait-mediated effects of tree neighbours (on leaf quality). Second, we show that tree diversity effects are contingent upon abiotic context (i.e. water stress). This observation is consistent with the recent finding that the relationships between forest biodiversity and ecosystem functioning are environmental context-dependent^43^. Third, our comparisons among tree composition treatments suggest that trait-mediated effects of neighbours on herbivores under mild vs. stressful conditions could partly result from differences in resource (e.g. light and water) acquisition and allocation by trees and on the way by which herbivores adapt their consumption and metabolism to their diet. Altogether, our results suggest that tree diversity affects patterns of insect herbivory in several different ways and that the mechanisms underlying such plant diversity effects on herbivores can be only entirely understood by simultaneously investigating the responses of plant functional traits, herbivore performance and feeding behaviour. In conclusion, our findings highlight the complexity of climate (e.g. drought) effects on tree-herbivores interactions and the need for proper manipulative experiments to feed predictive models.

## Materials and Methods

### Natural history

Silver birch (*Betula pendula*, Roth, Betulacea) is a broadleaved woody species widely distributed across Europe, from the Mediterranean area to central Siberia where it tolerates a large range of climatic and edaphic conditions^44^. It forms mature forests in Northern and Western Europe, while it is a common pioneer species in the central and southern parts of its geographical range where the present study was carried out. Leaf burst in this species usually occurs in early spring whereas leaf senescence and leaf drop typically starts in late summer. *Betula pendula* is attacked by a large community of insect herbivores, mainly generalist and specialist leaf chewers^44,45^ that may cause significant growth losses, even at low defoliation level.

The Gypsy moth (*Lymantria dispar* L., Lepidoptera) is a univoltine Lepidoptera (Lymantriidae) native to Europe and naturally present in the study area. *Lymantria dispar* is a generalist defoliator that principaly feeds on several broadleaved species and also on conifers. Although the principal host species varies across its geographic range, gypsy moth larvae have marked preferences for oaks and birches^46^.

### Experimental design

#### The ORPHEE experiment

We conducted this study at the ORPHEE tree diversity experiment in SW France (44°440N, 00°460W). It is a long-term experiment established in 2008 that aims at investigating the effects of tree diversity on forest ecosystem functioning. The experiment consists in eight blocks covering 12 ha, with 32 plots in every block corresponding to the 31 possible combinations of one to five tree species (*Betula pendula*, *Quercus robur*, *Q*. *pyrenaica*, *Q*. *ilex* and *Pinus pinaster*), with an additional replicate of the five-species plot. Each plot contains 10 rows of 10 trees planted 2 m apart (100 trees). Tree species mixtures were established according to a substitutive design, keeping tree density and the identity of tree neighbours equal across plots. Within plots, individual trees from different species were planted in a regular alternate pattern, such that a tree from a given species has at least one neighbour from each of the other species^5^.

In the present study, we focused on birch monocultures, two-species mixtures associating birch with pedunculate oak or pine, and the mixtures of pedunculate oak, pine and birch, for a total of 32 plots, corresponding to 4 plots × 8 blocks. These plots were chosen in order to span a large gradient of vertical heterogeneity and canopy closure and to associate birch with a broadleaved or a conifer species or both.

#### Irrigation treatment and water stress

From 2015 on, half of the blocks were irrigated from May to October while the other half only received natural rainfall. Mean annual precipitations and temperatures in the study area were 876.3 mm and 12.75 °C during the 1996–2016 period. The local climate is characterized by a chronic water deficit in summer, hence the non-irrigated blocks experience water stress during two to three month on average (mid-June to mid-September). Irrigation treatment consisted in sprinkling *ca* 42 m³ per night and per block, corresponding to *ca* 3 mm.night^−1^ per plot. This volume was calculated based on regional climatic data (evapotranspiration) and is assumed to avoid any soil water deficit in the irrigated blocks during the entire growing season^32^.

### Feeding trials with Gypsy moth larvae

We tested the effect of tree species composition and irrigation treatment on the performance of gypsy moth larvae by using laboratory feeding trials. We obtained first instar larvae from egg masses collected in early 2016 in an outbreak area of South East France. Neonate larvae were reared with leaves collected on a single oak tree (*Quercus robur*, near the ORPHEE experiment) for *ca* 20 days until they reached the third instar stage. This procedure ensured that all larvae were reared with the same substrate such that the comparison among irrigation and composition treatments was not biased by previous rearing conditions.

In May 2016, we randomly sampled three individual birch trees out of the 36 innermost trees of each of the 32 plots, resulting in a sample size of 96 trees (‘focal trees’ hereafter). We collected one branch with 50–100 leaves on each of the focal birch trees with clippers. Branches were pooled per Irrigation × Composition treatment, giving a total of 12 branches per treatment (1 branch × 3 birches × 4 replicates for each of the eight treatments). We stored branches in pots with water in a dark room maintained at 4 °C for the duration of the laboratory experiment. We isolated 96 third instar larvae into individual 20 × 29 × 5 cm plastic boxes for 24 h with no food and then weighed them to the closest µg. Twelve of these larvae were randomly assigned to each one of the eight Irrigation × Composition treatments consisting in one of the four specific compositions (B: Birch, BQ: Birch + Oak, BP: Birch + Pine, BQP: Birch + Oak + Pine), crossed with two levels of irrigation (Irrigated vs. Non-irrigated plots). The resulting design was a fully factorial experiment with 12 replicates per treatment.

Every morning, we randomly selected 24 fresh fully expanded and hardened birch leaves per treatment. Each caterpillar thus received two new fresh leaves a day, while partially consumed leaves were removed from rearing boxes. The experiment was carried out at room temperature (*ca* 20 °C) and lasted for eight days. Leaves introduced into each rearing box were scanned before and after consumption by Gypsy moth larvae. Small fragments of leaves were carefully isolated from frass and scanned together with leaves. Leaf surface was measured from scanned images using the software ImageJ. After scanning, consumed leaves from each replicate were transferred into paper bags, dried for 48 h at 55 °C and weighed.

We used the leaf area consumed to estimate herbivore food consumption^47^. At the end of the experiment, larvae were kept without food for another 24 h and weighed. Frass cumulated over the time of the experiment was dried at 55 °C for 48 h and weighed. Total leaf consumption (mg_leaf, dry weight_), larval growth (mg_larvae, fresh weight_) and total produced frass (mg_frass, dry weight_) were used in analyses of co-variance (ANCOVA) to test the interactive effects of composition × irrigation on relative larval growth rate and approximate birch leaf digestibility (see below and Fig. 4).

### Nutritional indices

#### Leaf biomass consumption

Leaf biomass consumption was estimated from leaf area consumed by Gypsy moth larvae^47^. For each treatment *i* (from 1 to 8) corresponding to the two irrigation and four composition treatments, the relationship between leaf surface (S) and leaf biomass (B) was used to estimate the amount of biomass consumed (ΔB_leaf,i_) from the surface removed by larvae (ΔS_leaf,i_). To establish these allometric relationships we used the remaining leaf surface at day + 1 (S_leaf,i,t+1_) and the corresponding biomass (dry weight, B_leaf,i,t+1_).

We first estimated parameters α_i_ and β_i_ of linear regressions for each treatment (Eq. 1):

1 $${{\rm{B}}}_{{\rm{leaf}},{\rm{i}},{\rm{t}}+1}={{\rm{\beta }}}_{{\rm{i}}}\times {{\rm{S}}}_{{\rm{leaf}},{\rm{i}},{\rm{t}}+1}+{{\rm{\alpha }}}_{{\rm{i}}}$$

We then used treatment-specific parameters α_i_ and β_i_ to estimate ΔB_leaf,i_ from ΔS_leaf,i_ (Eq. 2):

2 $${{\rm{\Delta }}{\rm{B}}}_{{\rm{leaf}},{\rm{i}}}={\rm{\beta }}{\rm{i}}\times {{\rm{\Delta }}{\rm{S}}}_{{\rm{leaf}},{\rm{i}}}+{\rm{\alpha }}{\rm{i}}$$

where ΔS_leaf,i_ = S_leaf,i,t_ − S_leaf,i,t+1_, that is the leaf area consumed by larvae between day *t* and *t* + 1.

#### Larval growth and nutritional indices

General approach: The classical approach consisting in calculating nutritional ratios^48^ such as relative growth rate (RGR) to compare diets with ANOVAs has several drawbacks that can be solved using analyses of covariance (ANCOVA), where final weight is the response variable, and initial weight a covariate^49^. There are three main reason why ANCOVAs perform better then ANOVAs when analysing this type of data^50,51^: (i) the ability to detect small differences between diets is greater when using ANCOVA, which is of particular interest when addressing intraspecific variability in plant quality; (ii) RGR fails to remove the effect of initial weight when both initial and final weight are significantly correlated, making ANOVA flawed; (iii) the analysis of RGR with ANOVA implicitly assumes that the effect of initial weight on growth is the same whatever the diet (*i*.*e*., no initial weight × diet interaction), which remains to be demonstrated. Larval growth and performance were thus analysed using the ANCOVA equivalent of Waldbauer’s indices^48^ (Fig. 4).

Larval growth (G, mg day^−1^): We analysed the effects of irrigation and tree species composition as well as all possible two-ways interactions on larval daily growth (*i*.*e*., final weight − initial weight, G = [*w*_*f*_ − *w*_*i*_]/duration of the experiment), with initial larval weight (*w*_*i*_,) as a covariate. This is the ANCOVA equivalent of Relative Growth Rate (RGR) index. Significant *w*_*i*_ × *Treatment* interactions (*Treatment* being Irrigation, Composition or their interaction) therefore indicate significant differences in RGR.

Leaf biomass consumption (ΔB, mg day^−1^): We analysed the effects of irrigation and tree species composition, as well as all possible two-ways interactions on leaf consumption (ΔB_leaf_, Eq. 2), with w_i_ as a covariate, which is the ANCOVA equivalent of Relative Consumption Rate (RCR) index. Significant w_i_ × Treatment interactions therefore indicate differences in RCR. In the absence of w_i_ × Treatment interactions, significant differences among treatments indicate that differences in consumed biomass were independent of caterpillar weight and thus resulted from pre-ingestive regulatory mechanisms.

Efficiency of conversion of ingested food into body mass (ECI, mg mg^−1^): Larval growth is regulated by both pre- and post-ingestive processes that can be approximated by accounting for leaf biomass consumption (ΔB, mg) and frass production (F, mg). We analysed the effects of biomass consumption, irrigation and tree species composition, as well as all possible two- and three-ways interactions on larval growth. ECI is therefore reflected by the slope of the regression of growth on leaf biomass consumption, which is the ANCOVA equivalent of Waldbauer’s ECI (G/ΔB). If differences in growth among treatments resulted only from pre-ingestive processes, there would be no effect of irrigation and composition treatments on growth once consumption is taken into account. Otherwise, pre- and post-ingestive processes may be involved. ECI informs on different pre-ingestive regulatory mechanisms among treatments and significant differences in ECI among treatments are detected by significant Consumption × Treatment interactions.

Efficiency of conversion of digested biomass (ECD, mg mg^−1^): Post-digestive mechanisms were detected by analysing the effects of irrigation, composition, digested biomass (i.e., consumed biomass – frass mass as a covariate) and all two- and three-ways interactions on larval growth. This analysis is the ANCOVA equivalent of Waldbauer’s ECD (G/[ΔB − F]). The disappearance of treatment effects on growth once the amount of digested food is taken into account as a covariate (i.e., ECD) would indicate that only pre-digestive processes are responsible for differences in growth among treatments. Otherwise, post-digestive (i.e., metabolic) processes may be involved as well. Therefore, differential larval growth among treatment is due to post-digestive mechanisms if there is a significant interaction between digestive biomass and treatments.

Approximate leaf digestibility (AD, mg mg^−1^): Mechanisms occurring after ingestion but prior to assimilation are detected by the differential amount of frass produced under different Irrigation × Composition treatments. We analysed the effects of irrigation, composition, leaf biomass consumption and all two- and three-ways interactions on frass production, which is the ANCOVA equivalent of Waldbauer’s AD (F/ΔB). We used AD as a proxy for leaf quality: the greater digestibility, the higher leaf quality. Digestibility differs among treatments if there is a significant Consumption × Treatment interaction. AD is given by the coefficient associated with the consumption parameter (*i*.*e*., regression slope). Coefficient values close to one indicate that most of ingested food is excreted (i.e., low digestibility indicating poor leaf quality).

### Statistical analyses

All analyses were done in *R*, version 3.3.1^52^, using base functions and the package *multcomp*^53^. We used linear models to analyse data from the laboratory experiment. For each response variable (G, ΔB, F), we first built a full model including the appropriate covariates, irrigation and tree species composition as factors, and all two-ways interactions. We then simplified the initial complete model by sequentially removing non-significant interactive terms, starting with the least significant term, before reporting model parameter estimates. When there was a significant Irrigation × Composition interaction, contrasts among composition treatments were estimated for each irrigation level separately, and contrasts between irrigation treatments were estimated for each composition separately. Normality and homogeneity of residuals were assessed graphically and response variables were log-transformed whenever necessary in order to satisfy model assumptions. In figures, we show raw data and raw means (±SE) rather than model predictions.

### Data availability

The dataset generated during the current study is available from the corresponding author on reasonable request.

## References

[1] Moreira X, Abdala-Roberts L, Rasmann S, Castagneyrol B, Mooney KA (2016). Plant diversity effects on insect herbivores and their natural enemies: current thinking, recent findings, and future directions. Curr. Opin. Insect Sci..

[2] Barbosa P (2009). Associational Resistance and Associational Susceptibility: Having Right or Wrong Neighbors. Annu Rev Ecol Evol Syst.

[3] Root RB (1973). Organization of a Plant-Arthropod Association in Simple and Diverse Habitats: The Fauna of Collards (*Brassica Oleracea*). Ecol. Monogr..

[4] Hambäck PA, Englund G (2005). Patch area, population density and the scaling of migration rates: the resource concentration hypothesis revisited: Density-area relations in sources and sinks. Ecol. Lett..

[5] Castagneyrol B, Giffard B, Péré C, Jactel H (2013). Plant apparency, an overlooked driver of associational resistance to insect herbivory. J. Ecol..

[6] Damien M (2016). Pest damage in mixed forests: Disentangling the effects of neighbor identity, host density and host apparency at different spatial scales. For. Ecol. Manag..

[7] Mraja A, Unsicker SB, Reichelt M, Gershenzon J, Roscher C (2011). Plant Community Diversity Influences Allocation to Direct Chemical Defence in Plantago lanceolata. PLoS ONE.

[8] Moreira X, Abdala-Roberts L, Parra-Tabla V, Mooney KA (2014). Positive Effects of Plant Genotypic and Species Diversity on Anti-Herbivore Defenses in a Tropical Tree Species. PLoS ONE.

[9] Glassmire AE (2016). Intraspecific phytochemical variation shapes community and population structure for specialist caterpillars. New Phytol..

[10] Moreira, X., Glauser, G. & Abdala-Roberts, L. Interactive effects of plant neighbourhood and ontogeny on insect herbivory and plant defensive traits. *Sci*. *Rep*. **7**, (2017). 10.1038/s41598-017-04314-3 PMC5481422 28642497

[11] Kostenko, O., Mulder, P. P. J., Courbois, M. & Bezemer, T. M. Effects of plant diversity on the concentration of secondary plant metabolites and the density of arthropods on focal plants in the field. *J*. *Ecol*. 10.1111/1365-2745.12700 (2016).

[12] Wäschke N, Hancock C, Hilker M, Obermaier E, Meiners T (2015). Does vegetation complexity affect host plant chemistry, and thus multitrophic interactions, in a human-altered landscape?. Oecologia.

[13] Kos M, Bukovinszky T, Mulder PPJ, Bezemer TM (2015). Disentangling above- and belowground neighbor effects on the growth, chemistry, and arthropod community on a focal plant. Ecology.

[14] Castagneyrol B, Jactel H (2012). Unraveling plant-animal diversity relationships: a meta-regression analysis. Ecology.

[15] Loranger J (2013). Predicting invertebrate herbivory from plant traits: Polycultures show strong nonadditive effects. Ecology.

[16] Muiruri EW, Milligan HT, Morath S, Koricheva J (2015). Moose browsing alters tree diversity effects on birch growth and insect herbivory. Funct. Ecol..

[17] Setiawan NN, Vanhellemont M, Baeten L, Dillen M, Verheyen K (2014). The effects of local neighbourhood diversity on pest and disease damage of trees in a young experimental forest. For. Ecol. Manag..

[18] Alalouni U, Brandl R, Auge H, Schädler M (2014). Does insect herbivory on oak depend on the diversity of tree stands?. Basic Appl. Ecol..

[19] Muiruri EW, Koricheva J (2016). Going undercover: increasing canopy cover around a host tree drives associational resistance to an insect pest. Oikos.

[20] White TC (1974). A hypothesis to explain outbreaks of looper caterpillars, with special reference to populations of *Selidosema suavis* in a plantation of *Pinus radiata* in New Zealand. Oecologia.

[21] Huberty AF, Denno RF (2004). Plant water stress and its consequences for herbivorous insects: a new synthesis. Ecology.

[22] Walter J (2011). How do extreme drought and plant community composition affect host plant metabolites and herbivore performance?. Arthropod-Plant Interact..

[23] Jactel H (2012). Drought effects on damage by forest insects and pathogens: a meta-analysis. Glob. Change Biol..

[24] Forrester DI, Theiveyanathan S, Collopy JJ, Marcar NE (2010). Enhanced water use efficiency in a mixed *Eucalyptus globulus* and *Acacia mearnsii* plantation. For. Ecol. Manag..

[25] Otieno D (2012). Drought responses of *Arrhenatherum elatius* grown in plant assemblages of varying species richness. Acta Oecologica-Int. J. Ecol..

[26] Jactel H (2017). Tree Diversity Drives Forest Stand Resistance to Natural Disturbances. Curr. For. Rep..

[27] Klaus VH (2016). Plant diversity moderates drought stress in grasslands: Implications from a large real-world study on 13C natural abundances. Sci. Total Environ..

[28] Mueller KE, Tilman D, Fornara DA, Hobbie SE (2013). Root depth distribution and the diversity–productivity relationship in a long-term grassland experiment. Ecology.

[29] Grettenberger IM, Tooker JF (2016). Inter-varietal interactions among plants in genotypically diverse mixtures tend to decrease herbivore performance. Oecologia.

[30] Kambach S, Kühn I, Castagneyrol B, Bruelheide H (2016). The Impact of Tree Diversity on Different Aspects of Insect Herbivory along a Global Temperature Gradient - A Meta-Analysis. Plos One.

[31] Roberts MR, Paul ND (2006). Seduced by the dark side: integrating molecular and ecological perspectives on the influence of light on plant defence against pests and pathogens. New Phytol..

[32] Castagneyrol B (2017). Bottom-up and top-down effects of tree species diversity on leaf insect herbivory. Ecol. Evol..

[33] Moreira, X., Mooney, K. A., Zas, R. & Sampedro, L. Bottom-up effects of host-plant species diversity and top-down effects of ants interactively increase plant performance. *Proc*. *R*. *Soc*. *Lond*. *B Biol*. *Sci*. rspb20120893, 10.1098/rspb.2012.0893 (2012). 10.1098/rspb.2012.0893 PMC3479792 22951745

[34] Karowe DN, Martin MM (1989). The effects of quantity and quality of diet nitrogen on the growth, efficiency of food utilization, nitrogen budget, and metabolic rate of fifth-instar Spodoptera eridania larvae (Lepidoptera: Noctuidae). J. Insect Physiol..

[35] Scott IM, Thaler JS, Scott JG (2010). Response of a Generalist Herbivore *Trichoplusia ni* to Jasmonate-Mediated Induced Defense in Tomato. J. Chem. Ecol..

[36] Huang, W., Zwimpfer, E., Hervé, M. R., Bont, Z. & Erb, M. Neighbourhood effects determine plant-herbivore interactions below-ground. *J*. *Ecol*. 10.1111/1365-2745.12805 (2017).

[37] Forey, E. *et al*. Tree species richness induces strong intraspecific variability of beech (Fagus sylvatica) leaf traits and alleviates edaphic stress. *Eur*. *J*. *For*. *Res*. 1–11 10.1007/s10342-016-0966-7 (2016).

[38] Hommel R (2016). Impact of interspecific competition and drought on the allocation of new assimilates in trees. Plant Biol..

[39] Castagneyrol, B., Jactel, H. & Moreira, X. Anti-herbivore defences and insect herbivory: Interactive effects of drought and tree neighbours. *J*. *Ecol*. 10.1111/1365-2745.12956 in press (2018).

[40] Perkins LE (2013). Generalist insects behave in a jasmonate-dependent manner on their host plants, leaving induced areas quickly and staying longer on distant parts. Proc. R. Soc. Lond. B Biol. Sci..

[41] Bucher R, Menzel F, Entling MH (2015). Risk of spider predation alters food web structure and reduces local herbivory in the field. Oecologia.

[42] Kaplan I, McArt SH, Thaler JS (2014). Plant Defenses and Predation Risk Differentially Shape Patterns of Consumption, Growth, and Digestive Efficiency in a Guild of Leaf-Chewing Insects. PLOS ONE.

[43] Ratcliffe S (2017). Biodiversity and ecosystem functioning relations in European forests depend on environmental context. Ecol. Lett..

[44] Atkinson MD (1992). Betula Pendula Roth (B. Verrucosa Ehrh.) and B. Pubescens Ehrh. J. Ecol..

[45] Kunca, A., Csoka, G. & Zubrik, M. Insects and diseases damaging trees and shrubs of Europe: a colour atlas. (2013).

[46] Mauffette Y, Lechowicz MJ, Jobin L (1983). Host preferences of the gypsy moth, *Lymantria dispar* (L.), in southern Quebec. Can. J. For. Res..

[47] Fernandez-Conradi, P., Jactel, H., Hampe, A., Leiva, M. J. & Castagneyrol, B. The effect of tree genetic diversity on insect herbivory varies with insect abundance. *Ecosphere* **8** (2017).

[48] Waldbauer GP (1968). The Consumption and Utilization of Food byInsects. Adv. Insect Physiol..

[49] Hägele BF, Rowell-Rahier M (1999). Dietary mixing in three generalist herbivores: nutrient complementation or toxin dilution?. Oecologia.

[50] Raubenheimer D, Simpson SL (1992). Analysis of covariance: an alternative to nutritional indices. Entomol. Exp. Appl..

[51] Horton DR, Redak RA (1993). Further comments on analysis of covariance in insect dietary studies. Entomol. Exp. Appl..

[52] R Core Team. *R: a language and environment for statistical computing*. (R fundation for statistical computing, 2016).

[53] Hothorn, T. *et al*. *multcomp: Simultaneous Inference in General Parametric Models*. (2016). 10.1002/bimj.200810425 18481363

